# Death of Dr. George Hayward

**Published:** 1863-10

**Authors:** 


DEATH OF DR. GEORGE HAYWARD.—While the meeting of the Councillors of the Massachusetts Medical Society was in session yesterday, Dr. John Jeffries announced the sudden decease, within an hour, of Dr. George Hayward, of this city, by apoplexy. The abrustness of this announcement produced a profound impression upon the gentlemen present, many of whom were among Dr. Hayward's old friends and associates. A committee was at once appointed to recommend some action on the part of the Councillors in view of this sad event.—*Boston Medical Journal.*

---

☞ The English Medical Act which compels every person practicing medicine and surgery to be qualified and registered does not, it seems, prevent quacks from this country obtaining a foothold in the profession.—Many of these graduates from chartered medical colleges exhibit their diplomas, are registered, and commence practice. The profession of England should understand that our State Legislatures charter colleges of every complexion, and the graduates of these institutions are therefore legally qualified. The title of M. D. in this country has no significance whatever. —*N. Y. Medical Times.*

